# The role of interoceptive awareness in social anxiety disorder – results of an internet-based CBT-manualized therapist delivered group-treatment

**DOI:** 10.1186/s40359-025-03013-3

**Published:** 2025-07-07

**Authors:** I. Schmalbach, K. Petrowski

**Affiliations:** Department of Medical Psychology and Medical Sociology, University Medical Centre of the Johannes Gutenberg-University Mainz, Mainz, Germany

**Keywords:** Therapist-delivered, Internet-based CBT group psychotherapy (ICBT), Interoception, Interoceptive awareness, Social anxiety disorder, Therapy outcome

## Abstract

**Background:**

Interoception, the process of detecting internal bodily signals, is linked to emotional regulation and mental health. Dysregulated interoceptive awareness (IAw) is associated with psychopathology, including social anxiety disorder (SAD), where internal cues influence negative self-perception and anxiety. While internet-based cognitive behavioral therapy (ICBT) offers an effective and more accessible treatment option, experimental treatment outcomes related to IAw and specific to SAD are rare. Hence, the present study aims to assess IAw and SAD symptom changes post-ICBT group therapy and determine whether IAw serves as a predictor of treatment outcome.

**Method:**

We collected data of *N* = 47 participants with SAD via online advertisement and outpatients of the University Hospital Clinic between November 2021 - May 2025. Participants met SAD criteria and underwent psychological assessments regarding IAw (via the Multidimensional Assessment of Interoceptive Awareness-2; MAIA-2) and symptoms of social anxiety (using the Liebowitz Social Anxiety Scale; LSAS) measured pre- and post-treatment. SAD participants received a three-month, therapist-delivered, internet-based CBT group psychotherapy (ICBT), focusing on behavioral experiments, psychoeducation, cognitive restructuring, and relapse prevention.

**Results:**

The study revealed significant reductions in SAD symptoms and improvements in interoceptive awareness dimensions as indicated by subscales of the MAIA-2 (i.e., Attention Regulation, Self-Regulation). Changes in Attention Regulation and Self-Regulation predicted a significant portion of variation in treatment outcome for SAD. Baseline values of IAw related to Not-Distracting and Not-Worrying also predicted treatment change. Combined predictors significantly predicted improvement in SAD symptomatology.

**Conclusion:**

This study provides strong evidence for the efficacy of therapist-delivered ICBT in reducing social anxiety symptoms while enhancing interoceptive capacities. Baseline Not-Distracting and Not-Worrying predicted better outcomes, highlighting their prognostic value. Improvements in Attention and Self-Regulation further contributed to symptom reduction, suggesting a key mechanism of action. These findings underscore the potential for interoceptive-focused interventions to optimize SAD treatment and highlight the accessibility of ICBT as a scalable therapeutic option.

## Background

Interoception, broadly defined, encompasses the processes of sensing, interpreting, and becoming aware of internal physiological signals—a capacity that is fundamental to self-awareness while playing a critical role in shaping emotional and behavioral responses [1–4]. These physiological signals originate not only from visceral systems (e.g., cardiovascular, respiratory, gastrointestinal systems) but also from cutaneous sensations such as affective touch and pain [1]–[2]. Given its inherent complexity, the conceptualization of interoception has evolved considerably over time. Once viewed merely as a homeostatic mechanism [5], it is now understood as a dynamic and multifaceted process that extends beyond basic physiological signals [6–8]. In this context, one influential model delineates three dimensions of interoceptive abilities: interoceptive accuracy (IAcc), interoceptive sensibility (IS), and interoceptive awareness (IAw) [7, 9]. *Interoceptive accuracy* (or sensitivity) [7] refers to the objective ability to correctly detect internal bodily signals—for example, perceiving one’s own heartbeat [7, 10]. In contrast, *interoceptive awareness* denotes metacognitive insight into the accuracy of these detections, i.e., the extent to which interoceptive judgments align with actual performance [9]. Notably, this notion differs from the broader psychological construct of metacognitive awareness as used in cognitive behavioral therapies [11, 12]. Meanwhile, *interoceptive sensibility* reflects a self-evaluated assessment of subjective interoception, encompassing beliefs, attitudes, and the propensity to attend to bodily sensations - typically assessed via self-report scales [8]. In summary, while interoceptive awareness and accuracy emphasize the objective and metacognitive aspects of bodily perception, interoceptive sensibility highlights the subjective experience of these internal signals—a distinction with significant implications for mental health.

Interoceptive sensibility encompasses not only the detection of bodily sensations but also their interpretation, appraisal, and regulation [7, 13]. This subjective dimension is relevant to anxiety-related conditions [14, 15] as it helps distinguish between anxiety-driven and adaptive attentional styles—an essential distinction for achieving positive therapeutic outcomes. Anxiety-driven attention is associated with anxiety disorders, whereas adaptive attention promotes resilience and emotional regulation [8, 16–18]. In this context, the conceptual framework proposed by Mehling et al. [13, 17] is especially pertinent. Their self-report tool, the Multidimensional Assessment of Interoceptive Awareness (MAIA) distinguishes maladaptive attentional biases (e.g., distracting and worrying) from adaptive attention styles (e.g., non-judgmental awareness, trust) reflecting a dynamic interplay of regulatory processes. The MAIA includes the ability to *Notice* subtle bodily sensations, engage mindfully by *Not-Distracting* oneself from discomfort, and reduce the tendency to worry about such sensations (*Not-Worrying*). This regulatory facet suggests that an adaptive interoceptive style is characterized by a lower propensity to negatively appraise bodily signals, in contrast to maladaptive coping (e.g., worrying and distraction) [17]. Furthermore, the capacity to intentionally direct and sustain attention toward internal sensations—captured by dimensions such as *Attention Regulation* and *Emotional Awareness*—facilitates a deeper understanding of bodily states and emotions. Building on this, *Self-Regulation* refers to the ability to use interoceptive insights to manage psychological distress, thereby contributing to coping and resilience. Additionally, a rich interoceptive sensibility includes *Listening to the Body*, or engaging with bodily signals to guide behavior and decision-making, ultimately culminating in *Trusting*—confidence in one’s bodily sensations as a reliable source of internal information.

Dysregulated interoception has been linked to various psychopathologies [19, 20] including anxiety disorders [9, 21–23]. In the context of interoceptive sensibility, ignoring or distracting oneself from anxiety-related bodily sensations can be viewed as a form of avoidance. Although this strategy may temporarily alleviate distress, it can be maladaptive, as it interferes with the development of effective coping mechanisms [17]. Given that SAD is characterized by excessive self-focused attention [24, 25], understanding how interoceptive processes contribute to symptoms is essential. Despite its relevance, the role of interoception in SAD remains relatively underexplored. Individuals with SAD frequently rely on interoceptive cues—such as blushing or trembling—to infer how they are perceived by others. This tendency reflects a cognitive process termed “processing the self as a social object” [24]. Heightened self-monitoring of bodily signals contributes to distorted self-perceptions and maladaptive appraisals, thereby reinforcing symptoms and perpetuating cycles of negative affect [25, 26]. Moreover, attentional biases toward interoceptive signals play a critical role in maintaining SAD by exacerbating hypervigilance and self-focused attention [27, 28].

Despite growing recognition of interception’s relevance to anxiety disorders, empirical findings on its relationship with SAD remain inconsistent [29–31] even in non-clinical populations [32]. Slotta et al. [33] for example, identified a relationship between interoceptive sensibility and trait anxiety. In contrast, other studies propose that specific interoceptive dimensions—such as “Not Worrying” and “Self-Regulation”—may be inversely related to anxiety and potentially serve as protective factors [8, 29]. These conflicting findings underscore the complexity of interoceptive mechanisms in SAD and the need for further research to elucidate how distinct interoceptive dimensions influence anxiety symptomatology.

Considering the characteristic features of SAD (e.g., excessive self-focused attention), it is crucial to explore therapeutic strategies that target dysfunctional interoceptive processes. Cognitive behavioral therapy (CBT) as a well-established treatment for SAD, aims to correct maladaptive patterns that reinforce worry and negative interpretations of bodily sensations (e.g., sweating or blushing) [24, 34]. Because CBT effectively reduces emotional dysregulation, it may also modulate interoceptive sensibility by fostering adaptive attention to and interpretation of bodily sensations [29, 35]. Techniques such as cognitive restructuring, attention training, and exposure may strength interoceptive domains like “Not Worrying” and “Self- and Attention Regulation,” thereby promoting adaptive responses [35, 36]. Although CBT is effective, its accessibility is often limited by stigma, logistical challenges, cost, and availability [37–39]. As an alternative, internet-based CBT (ICBT) has emerged as a more cost-effective and accessible option that can address many of these barriers [40]. In particular, group-based ICBT offers interactional support [41–43] within participants’ daily environment. Despite its potential, little is known about the impact of group-ICBT’s on interoceptive sensibility in individuals with SAD.

This study addresses this gap by investigating the role of interoceptive sensibility in SAD. Specifically, we examine pre- to post-treatment changes in interoceptive dimensions, as measured by the MAIA-2, and social anxiety symptoms, assessed by using the LSAS. Our primary objective is to explore the predictive value of baseline interoceptive dimensions and their changes during treatment – for therapeutic outcomes, particularly in the context of group-based ICBT.

## Method

This manuscript presents exploratory data from an experimental repeated-measures study (within-subjects, pre-post design) funded by the Deutsche Forschungsgemeinschaft (Project number: GZ: PE 1804/18 − 1) investigating therapy outcomes in individuals with SAD before and after ICBT. The current analysis specifically examines interoceptive sensibility as measured by the MAIA-2 questionnaire, utilizing data collected between November 2021 and May 2025. Importantly, this represents an analysis of the measures collected during this timeframe, with no selective outcome reporting. The findings presented in this manuscript constitute the first publication from this longitudinal investigation. The research questions and analyses presented here were planned as part of the original study protocol to address a specific aspect (i.e., interoception) of the overall research program. The study sample comprised three outpatient participants (*n* = 3) from the University Hospital Clinic and Polyclinic for Psychosomatic Medicine and Psychotherapy in Mainz, while the majority of participants were recruited through online advertisements. Eligible participants who met the diagnostic criteria for SAD during the initial structured interview (DIPS) [44] and scored at or above the cutoff (LSAS total score ≥ 30) on the disorder-specific questionnaire where eligible. However, participants where included in the study if they met the inclusion criteria (age between 18 and 65 years, primary diagnosis of social phobia, no comorbid disorders such as substance use or major depression disorder; ICD-10 [45]: F32.2 – F32.3). A comorbid diagnosis of mild depression (ICD-10: F32.1) did not exclude participation due to its high comorbidity with SAD [46]. Given the experimental design, a strictly homogeneous sample was essential for gaining a comprehensive understanding of individuals with SAD minimizing the potential influence of confounding variables. The participants were consecutively recruited and accommodated in a treatment group resulting in a sample of *N* = 47 participants. Detailed patient characteristics are presented in Table 1. All study participants were thoroughly informed about the procedures and gave written consent before participation. The study protocol was approved by the local Ethics Committee of the University Hospital Carl Gustav Carus Dresden (Approval No. 44022014).

**Table 1:** Characteristics of the participants

	Total Sample (*N* = 47)
Female. *n* (%)	33 (70%)
Age in yrs. *M (SD)*	31 (9.9)
German. *n (%)*	46 (98%)
Relationship status	
Single	38 (81%)
Married	9 (19%)
*Qualification. *n (%)*	
University diploma	9 (19%)
High school diploma	27 (57%)
Apprenticeship	6 (3%)
Specialized secondary school diploma	3 (6%)
9th Grade	1 (2%)

Note. *The German education system. a high school diploma qualifies for university admission

While the diploma until the 8–10th grade qualifies for vocational training

### Psychopathological assessments

The *Liebowitz Social Anxiety Scale* (LSAS) [47] is a widely recognized scale used to assess social anxiety disorder, measuring both fear and avoidance across a range of social situations. It consists of 24 items − 13 social and 11 focused on performance-based situations (e.g., “telephoning in public “; “participating in small groups”)- which respondents rate based on their experiences of fear and avoidance in specific social contexts. Items are scored from 0 (“never”) to 3 (“always”) in relation to fear and avoidance. One key indicator of construct validity is the strong correlation between the LSAS and other established measures of social anxiety [48, 49]. For instance, the LSAS demonstrates a high correlation with the clinician-administered Liebowitz Social Anxiety Scale [50]. The LSAS has excellent psychometric properties [51] and is widely used with high internal consistency (Cronbach’s α =.95–.97) [51–53].

The *Multidimensional Assessment of Interoceptive Awareness* (MAIA-2) [13] is a 32-item self-report instrument rated on a 6-point Likert scale, developed to distinguish between adaptive and maladaptive styles of interoceptive attention [8, 17]. Participants indicate how frequently each statement applies to their daily experience, with responses options ranging from 0 (“never”) to 5 (“always”). The MAIA-2 assesses eight different dimensions: Noticing, Not-Distracting, Not-Worrying, Attention Regulation, Self-Regulation, Body Listening and Trusting. Scores are calculated separately for each subscale, rather than yielding a global score. The MAIA is a well-validated and reliable instrument for a comprehensive assessment [13], with prior studies reporting internal consistency coefficients around α = 0.83 [18, 54–56]. Regarding construct validity the subscales demonstrates distinct relationships with anxiety measures. *Emotional Awareness* shows weak associations with trait anxiety, whereas subscales such as Attention *Regulation*, *Not-Worrying*, *and Trusting* exhibit stronger negative correlations [16]. For example, a German study found that Noticing and Body Listening were minimally related to trait anxiety (*r* = −.10 to 0.10), whereas higher Attention Regulation and Trusting scores correlated with lower state anxiety, especially among individuals with elevated anxiety levels [57]. Overall, the MAIA reflects a departure from traditional views of interoception as maladaptive bodily awareness, which has historically been linked to adverse health outcomes [58].

### Treatment

Psychometric assessments were administered before and after a structured internet-based group intervention. The treatment consisted of a three-month, manualized cognitive behavioral therapy program [24, 34], adapted for online delivery in accordance with established protocols [59–61]. The sessions were conducted weekly via videoconference, each lasting 3.5 h, with a maximum of six participants per group. The intervention was delivered by licensed psychologists or supervised psychological psychotherapists in training. The intervention comprised five core components: (1) development of an individualized explanatory model, (2) attention training, (3) behavioral experiments (including online exposure), (4) cognitive restructuring techniques, and (5) relapse prevention. Behavioral experiments—such as in-situ exercises and video feedback—formed a central part of the treatment, aiming to test and challenge participants’ core beliefs. Additionally, participants were assigned homework involving real-life exposure tasks designed to target maladaptive beliefs and enhance their capacity to cope with anxiety-provoking situations.

### Statistical analysis

All statistical analyses were conducted using *R* version 4.2.2 (R Development Core Team) [62]. The power analysis for this study was conducted with Stata/MP 14 [63] and refers to the primary study mentioned above. Specifically, the mean difference was estimated using the data reported by Guo et al. [40] which provided an empirical basis for our calculations. Given a moderate effect size (*d* = 0.55), an alpha level of 0.05, and a power of 80%, the required sample size was *N* = 28 for a repeated measures *t*-test. Accounting for 10% missing data, the target sample size was adjusted to *n* = 31 participants. In addition, for the regression analysis assessing the predictive role of interoceptive dimensions on treatment outcomes, we assumed, based on prior research based on post-treatment anxiety outcomes [8, 18, 64] a correlation of *r* =.40 between changes in interoceptive sensibility and changes in social anxiety disorder symptoms. With an alpha level of 0.05 and a power of 80%, this required a minimum of 46 participants. Considering a 10% rate of missing data, the final target sample size was adjusted to *n* = 51 participants.

Pre-post changes in symptoms related to SAD (LSAS) and interoceptive sensibility (MAIA-2) were determined via paired samples t-tests. To further explore the predictive capacity of baseline interoceptive values and changes in interoception on treatment outcome, we employed a two-stage regression approach. In the first stage, we conducted bivariate linear regression analyses for each of the eight subscales of the MAIA-2 at baseline, as well as the pre-to-post treatment change scores for each MAIA-2 subscale, to individually predict treatment outcome, which was operationalized as the difference in LSAS total scores before and after treatment. This initial step allowed us to identify individual interoceptive dimensions that demonstrated a statistically significant relationship with treatment outcome based on a significance level of *p* < 0.05. In the second stage, to assess the combined predictive power of the interoceptive dimensions identified as significant in the bivariate analyses, and to account for potential shared variance among these predictors, we constructed a multiple linear regression model. This multivariate model, referred to in the results section as a joint model, included only those baseline MAIA-2 subscales and MAIA-2 change scores that had emerged as significant predictors of treatment outcome in the preceding bivariate regression analyses. The primary rationale for this approach was to move beyond examining isolated relationships and to understand how these significant interoceptive dimensions collectively contribute to the prediction of treatment outcome while controlling for their intercorrelations. By simultaneously entering these predictors into a single model, we aimed to: 1. Determine the overall proportion of variance explained, as indicated by the adjusted R-squared value of the model. 2. Account for shared variance and identify unique contributions: Given that some dimensions of interoceptive awareness may be correlated (as explored in the correlation matrix presented in Table 2), this process essentially ‘partials out’ the shared variance, providing a more accurate understanding of each predictor’s independent effect. 3. Identify primary and independent predictors: By examining the regression coefficients (beta weights) associated with each predictor in the joint model, we could assess the relative strength and direction of their independent effects on treatment outcome while controlling for the influence of the other significant interoceptive dimensions included in the model.

**Table 2:** Correlation matrix of the interoception and social anxiety scales

	1	2	3	4	5	6	7	8	9	10	11
1 - Noticing	1										
2 - Not-Distracting	− 0.20*	1									
3 - Not-Worrying	0.11	− 0.14	1								
4 - Attention-Regulation	0.56*	− 0.07	0.29*	1							
5 - Emotional Awareness	0.65*	− 0.10	0.09	0.57*	1						
6 - Self-Regulation	0.60*	− 0.18*	0.16*	0.74*	0.65*	1					
7 - Body-Listening	0.52*	− 0.13	0.10	0.61*	0.54*	0.63*	1				
8 - Trust	0.23*	− 0.05	0.18*	0.45*	0.38*	0.42*	0.39*	1			
9 – LSAS SI	− 0.08	− 0.11	− 0.13	− 0.28*	− 0.08	− 0.24*	− 0.20*	− 0.12	1		
10 – LSAS SA	− 0.00	− 0.11	− 0.11	− 0.28*	− 0.02	− 0.21*	− 0.21*	− 0.15	0.94*	1	
11 – LSAS Total	− 0.04	− 0.12	− 0.12	− 0.29*	− 0.05	− 0.23*	− 0.22*	− 0.14	0.98*	0.99*	1

Note. Correlations that are significant at 0.05 are marked with *

## Results

### Treatment outcome

Our data revealed significant reductions with large effect sizes in both global and specific symptoms of social anxiety (*d* = 0.92), including interactional (*d* = 0.97) and performance anxiety (*d* = 0.76). Similarly, significant changes were observed in specific dimensions of interoceptive awareness: Attention Regulation (*d* = 0.39) and Self-Regulation (*d* = 0.42). See Table 3 for further details.

**Table 3:** Interoceptive awareness and symptoms of social anxiety disorder - Pre-post comparisons

	ω	t1		t2		Paired t-tests
		M	SD	M	SD	
Noticing	0.78	3.86	0.91	3.72	0.84	*t*(39) = 1.68, *p* =.1, *d* = 0.27
Not-Distracting	0.92	3.58	0.98	3.39	0.85	*t*(39) = 1.21, *p* =.232, *d* = 0.19
Not-Worrying	0.85	3.35	0.86	3.49	0.87	*t*(39) = 1.45, *p* =.156, *d* = 0.23
Attention-Regulation	0.95	3.02	0.91	3.20	1.00	*t*(39) = 2.46, *p* =.018, *d* = 0.39
Emotional Awareness	0.86	4.34	0.92	4.14	0.96	*t*(39) = 0.57, *p* =.571, *d* = 0.09
Self-Regulation	0.85	3.27	1.01	3.55	1.02	*t*(39) = 2.69, *p* =.011, *d* = 0.42
Body-Listening	0.76	3.24	0.98	3.37	0.99	*t*(39) = 2.01, *p* =.051, *d* = 0.32
Trust	0.80	3.71	1.16	3.82	1.17	*t*(39) = 0.94, *p* =.354, *d* = 0.15
LSAS-SA	0.97	2.40	0.56	2.24	0.53	*t*(46) = 5.21, *p* <.001, *d* = 0.76
LSAS-SI	0.96	2.19	0.52	1.96	0.49	*t*(46) = 6.66, *p* <.001, *d* = 0.97
LSAS Total	0.98	2.30	0.53	2.10	0.50	*t*(46) = 6.31, *p* <.001, *d* = 0.92

Note. LSAS = Liebowitz Social Anxiety Scale (LSAS). Liebowitz Social Anxiety Scale (SI = Social Interaction). Liebowitz Social Anxiety Scale (SA = Performance). ω = Reliability coefficient

### Prediction of treatment outcome

Attention Regulation and Self-Regulation, were significant negative predictors of treatment outcomes related to social anxiety. In a joint model, these two dimensions (Attention-Regulation, Self-Regulation) predicted a significant portion of variation in treatment outcome: F(2,37) = 4.399, *p* =.019, R² = 0.192 (Adjusted R² = 0.150) – see Table 4. Conversely, baseline interoceptive values were predictive of changes in treatment outcomes only for the dimensions of Not-Distracting and Not-Worrying (see Table 4). In a joint model, these two dimensions (ND, NW) predicted a significant portion of variation in treatment outcome *F*(2,37) = 5.64, *p* =.007, *R²* = 0.233 (Adjusted *R²* = 0.192).

**Table 4:** Linear regression of changes in social anxiety disorder (LSAS)

	b	SE	*p*	*R* ^2^
Predictors at baseline				
Noticing	− 0.015	0.051	0.771	0.002
Not-Distracting	− 0.100	0.046	0.036	0.111
Not-Worrying	0.159	0.055	0.007	0.178
Attention-Regulation	− 0.044	0.051	0.396	0.019
Emotional Awareness	− 0.064	0.043	0.145	0.055
Self-Regulation	− 0.028	0.043	0.525	0.011
Body-Listening	0.005	0.045	0.904	0.000
Trust	0.002	0.039	0.965	0.000
Predictors changes				
Noticing	− 0.070	0.062	0.263	0.033
Not-Distracting	− 0.081	0.057	0.162	0.051
Not-Worrying	0.037	0.074	0.620	0.007
Attention-Regulation	− 0.133	0.048	0.008	0.169
Emotional Awareness	− 0.087	0.057	0.135	0.058
Self-Regulation	− 0.129	0.046	0.008	0.170
Body-Listening	− 0.002	0.064	0.980	0.000
Trust	− 0.022	0.052	0.679	0.005

Note. IN = Interoception total score

In the final multiple regression model, all four of the above mentioned significant predictors (Attention Regulation, Self-Regulation, Not-Distracting and Not-Worrying) combined yielded: *F*(4,35) = 4.48, *p* =.004, *R²* = 0.339 (Adjusted *R²* = 0.265), indicating a significant model. This suggests that the pre-post measured improvement can be significantly predicted by these interoceptive dimensions. To rule out potential influences of collinearity on the analyses, we calculated the variance inflation factors (*VIF*) for all eight predictors across the three models. *VIF* was between 1.05 and 2.17 with an average value of 1.58. We thus conclude that there are only negligible amounts collinearity in the analysis. To contextualize the regressions, we added a correlation matrix (see Table 2).

## Discussion

This study aimed to determine the relationship between interoceptive sensibility and SAD within the context of a three-month, therapist-delivered, ICBT group treatment. Specifically, we assessed pre- to post-treatment changes in both interoceptive dimensions and social anxiety symptoms. Furthermore, we explored the predictive value of specific interoceptive dimensions at baseline and their changes during treatment in relation to therapeutic outcomes. In sum, our findings revealed significant reductions in both global and specific symptoms of social anxiety following the ICBT intervention. This is consistent with its established efficacy [40, 41], while also highlighting the potential of group-format interventions delivered online [41, 42]. Importantly, we observed significant changes in specific domains of interoceptive sensibility, underlining the relevance of a multi-dimensional approach, as previously proposed [8, 9, 13]. In particular, *Attention Regulation* and *Self-Regulation* showed significant improvements post-treatment. Furthermore, our investigation revealed that baseline levels of *Not-Distracting* and *Not-Worrying* significantly predicted change in social anxiety symptoms, while changes in *Attention Regulation* and *Self-Regulation* during treatment also emerged as significant predictors of therapeutic outcome.

In detail, our findings revealed significant reductions in social anxiety symptoms across all LSAS scales following ICBT. Total LSAS scores decreased significantly, reflecting a large effect size. Similarly, both the social interaction and performance anxiety subcomponents showed significant reductions, each demonstrating large effect sizes. These results are consistent with previous meta-analytic findings related on the effects of group CBT and ICBT in alleviating symptoms of social anxiety [40–42]. Taking into account the characteristics of SAD and its treatment barriers, ICBT integration into participants’ daily routines emerges as a viable and useful treatment modality [59, 61]. In particular, our ICBT model combined professional guidance with peer support, fostering a sense of community while maintaining accessibility. These findings underscore the potential of ICBT as a scalable modality for addressing the treatment gap associated with SAD [65].

Regarding interoceptive sensibility, paired samples t-tests indicated significant improvements in specific domains of the MAIA-2: *Attention Regulation* and *Self-Regulation*. These moderate effect sizes suggest that participants developed a greater ability to sustain attention to internal bodily signals and to use interoceptive information for emotional regulation post ICBT. Notably, other dimensions (*Noticing*, *Not-Distracting*, *Not-Worrying*, *Emotional Awareness*, *Body-Listening*, *and Trust*) did not show statistically significant pre-post changes, indicating the domain-specific impact of ICBT on interoceptive sensibility.

Regression analyses further elucidated the predictive role of interoceptive sensibility in SAD treatment outcomes. The baseline scores of the subscale *Not-Distracting* significantly predicted reductions in LSAS scores, suggesting that individuals with lower initial *Not-Distracting* scores—indicative of a greater tendency to avoid bodily signals—experienced greater improvement in social anxiety symptoms. Distraction, a maladaptive avoidance strategy [8, 17], may provide temporary relief via negative reinforcement [24, 34] but impedes development of adaptive coping mechanisms. Our finding indicates that those participants with lower capacity in *not-distraction* at treatment onset might have a greater potential for therapeutic gain, specifically within this domain - as ICBT actively encourages a direct confrontation with bodily sensations (e.g., blushing). This pattern of results coincides with those observed by Borneman et al. [18], who found that lower baseline interoception scores predicted better post-treatment outcomes in a mindfulness-based intervention. They also reported that participants with low interoception scores at baseline exhibited enhanced post-treatment outcomes. The fact that *Not-Distracting* scores significantly predicted greater symptom reduction in our study, further emphasized its potential role as a prognostic factor in SAD treatment. Similarly, baseline values in *Not-Worrying* significantly predicted reduction in social anxiety symptoms following the ICBT intervention. According to the items of this scale it is suggested that individuals with higher baseline *Not-Worrying* scores may worry less in terms of bodily signals, such as pain or not feeling well. At the same time, it may also mean that individuals entering treatment with a higher baseline level of *Not-Worrying* are better positioned to leverage the techniques included in ICBT. Several potential mechanisms may underpin this predictive relationship. A higher baseline in *Not-Worrying* may serve as a proxy for lower anxiety, particularly concerning the fear of physiological arousal and its perceived negative consequences [66], thereby facilitating challenges linked to anxiety-driven thoughts associated with bodily signals and be more receptive to cognitive restructuring elements of ICBT, a cornerstone of SAD treatment [24, 34]. On the other hand, given that our participants exhibited a tendency toward distraction, these findings may as well suggest the use of thought suppression—a well-documented maladaptive strategy for managing worry in individuals with anxiety disorders [65, 67]. This strategy may temporarily reduce anxiety by preventing conscious engagement with distressing thoughts. Collectively, our data suggest that baseline tendencies toward distraction and worry about bodily sensations may serve as prognostic indicator for ICBT outcomes in SAD. However, these findings warrant further exploration.

Additionally, changes in interoceptive dimensions during treatment emerged as significant predictors of reductions in LSAS scores. Increases in both *Attention Regulation* and *Self-Regulation* were strongly associated with reductions in social anxiety symptoms, suggesting improved capacity for distress modulation. The observed improvements in *Attention Regulation and Self-Regulation* are consistent with prior evidence emphasizing the role of adaptive attentional and regulatory skills in mitigating hypervigilance and emotional dysregulation in anxiety disorders [13, 29]. Our results mirror those of Bornemann et al. [18], who found that the most pronounced treatment-related changes occurred in regulatory aspects of interoceptive sensibility—specifically, the extent to which bodily signals are utilized for self-regulation in daily life. Additionally, our results support the presence of a distinct pattern of effects across different facets of interoceptive sensibility.

Beyond the reported findings, our study provides novel insights into the potential mechanisms through which interoceptive changes contribute to therapeutic benefit. The observed improvements in *Attention Regulation and Self-Regulation* are consistent with evidence emphasizing the importance of adaptive attentional and regulatory capacities in mitigating hypervigilance and emotional dysregulation in anxiety disorders [23, 35]. Further, our findings help reconcile previous inconsistencies by highlighting dimensional specificity of interoceptive sensibility and its differential impact on treatment outcomes, as proposed by Mehling et al. [13]. Prior studies have shown mixed associations between interoception and anxiety; with some reporting positive correlations [3, 30], while others noted negative correlations for dimensions like *Not-Worrying* and *Self-Regulation* [8, 29]. The current study adds clarity by demonstrating that specific interoceptive dimensions predict distinct treatment outcomes (e.g. adaptive engagement of *Attention Regulation* and *Self-Regulation*). Conversely, *Not-Distracting* and *Not-Worrying*, predicted baseline treatment outcomes, suggesting a potential prognostic indicator in SAD treatment. These findings align with Mehling et al.‘s [16, 17] conceptualization of interoceptive sensibility as a multidimensional construct.

In general, these outcomes contribute to a better understanding of the relationship between interoception sensibility and SAD, a disorder that uniquely involves reliance on bodily cues to infer others’ judgments – an aspect that has remained underexplored. Specifically, our data suggested that improvements in *Attention Regulation* and *Self-Regulation* may help modulate excessive self-monitoring—a hallmark of SAD [24, 34]. The observed predictive role of *Not-Worrying* and *Not-Distracting* provides further clarifies how interoceptive dimensions interact with attentional and regulatory processes during therapy. By incorporating interoceptive-focused metrics into ICBT, this study supports both interoception research and clinical practice by broadening the scope of measurable therapeutic outcomes in SAD. Finally, these findings contribute to a growing body of evidence emphasizing the relevance of interoception in anxiety and other affective disorders [68, 69].

In summary, this study provides novel insights into the relationship between interoception and SAD within the context of ICBT. By identifying interoceptive dimensions as predictors of therapeutic outcomes, the findings suggest a potentially prognostic tool in social anxiety disorder. Based on our findings certain implications emerge: significant reductions in SAD symptoms alongside improvements in interoceptive sensibility, support the utility of ICBT in addressing both cognitive and certain components of interoceptive sensibility. For example, patients learned to manage anxiety-related physiological cues such as sweating, blushing and trembling through adaptive coping. By integrating multidimensional metrics of interoceptive sensibility into CBT may allow therapists to better tailor interventions to individual needs. For example, by incorporating “add-ons” during treatment to enhance self-regulation or improve attention regulation skills. The predictive role of *Not-Worrying* also suggests that fostering a non-catastrophic stance toward bodily sensations could enhance treatment engagement. Techniques such as cognitive restructuring and interoceptive exposure may help patients reframe bodily cues more adaptively. Improvements in *Attention Regulation* and *Self-Regulation* indicate that strengthening these skills during therapy can increase distress tolerance and emotional regulation. The scalability and accessibility of ICBT further enhance its clinical utility, especially for underserved populations where barriers to in-person therapy persist.

The present study is the first to examine interoceptive sensibility within a CBT-based protocol for SAD, yet several limitations must be acknowledged. First, the reliance on self-report measures for interoceptive dimensions and SAD symptoms introduces potential biases, including social desirability and subjective misjudgment. Future research should incorporate objective interoceptive assessments (e.g., heartbeat perception tasks) to enhance validity. Second, the absence of a control group limits causal inferences regarding ICBT’s specific effects on interoception. Randomized controlled trials comparing ICBT with alternative treatments are needed for definitive efficacy assessments. Additionally, future studies should examine whether targeted interoceptive training (e.g., mindfulness, interoceptive exposure) enhances ICBT outcomes and whether psychoeducation on interoceptive sensibility (e.g., adaptive body listening) before treatment optimizes therapeutic gains. Third, although the final sample size fell slightly short of the planned recruitment target, it remained sufficient to detect the expected effects and did not compromise the robustness of the primary findings. Nonetheless, the marginally reduced power may have limited the detection of smaller or more complex effects. Additionally, the use of online recruitment and the presence of mild comorbid depressive symptoms in the sample may constrain the generalizability of the results. Future research should aim to replicate these findings in more diverse clinical populations, including those with varying comorbidity profiles and recruitment contexts. Lastly, given interception’s transdiagnostic relevance, future studies should investigate whether interoceptive changes observed in SAD extend to other anxiety disorders (e.g., GAD, panic disorder), thereby broadening the clinical applicability of interoceptive-focused interventions.

## Conclusion

This study provides strong evidence for the efficacy of therapist-delivered ICBT in alleviating social anxiety symptoms while enhancing interoceptive capacities. Baseline Not-Distracting and Not-Worrying scores predicted more favorable outcomes, highlighting their prognostic value. Improvements in Attention and Self-Regulation were also associated with symptom reduction, suggesting a key therapeutic mechanism. These findings underscore the potential for interoceptive-focused interventions to optimize SAD treatment and reinforce the scalability and accessibility of ICBT as a viable therapeutic option.

## Data Availability

Availability of data and materials: The data that support the findings of this study are available from Petrowski Katja kpetrows@uni-mainz.de but restrictions apply to the availability of these data, which were used under licence for the current study and so are not publicly available. The data are, however, available from the authors upon reasonable request and with the of Katja Petrowski: kpetrows@uni-mainz.de.
